# Supplementary material for: Artificial neural network with Taguchi method for robust classification model to improve classification accuracy of breast cancer
Source: PeerJ Comput Sci. 2021 Jan 25;7:e344. doi: 10.7717/peerj-cs.344 (PMC7924699; doi:10.7717/peerj-cs.344)
Supplement: Supplemental Information 1 [file peerj-cs-07-344-s001.docx]

**Matlab Coding for Artificial Neural Network**

% Solve a Pattern Recognition Problem with a Neural Network

% Script generated by Neural Pattern Recognition app

% Created 04-Dec-2018 22:03:19

%

% input - input data.

% target - target data.

x = input;

t = target;

% Choose a Training Function

% For a list of all training functions type: help nntrain

% 'trainlm' is usually fastest.

% 'trainbr' takes longer but may be better for challenging problems.

% 'trainscg' uses less memory. Suitable in low memory situations.

trainFcn = 'trainscg'; % Scaled conjugate gradient backpropagation.

% Create a Pattern Recognition Network

hiddenLayerSize = 15;

net = patternnet(hiddenLayerSize, trainFcn);

% Choose Input and Output Pre/Post-Processing Functions

% For a list of all processing functions type: help nnprocess

net.input.processFcns = {'removeconstantrows','mapminmax'};

% Setup Division of Data for Training, Validation, Testing

% For a list of all data division functions type: help nndivision

net.divideFcn = 'dividerand'; % Divide data randomly

net.divideMode = 'sample'; % Divide up every sample

net.divideParam.trainRatio = 70/100;

net.divideParam.valRatio = 15/100;

net.divideParam.testRatio = 15/100;

% Choose a Performance Function

% For a list of all performance functions type: help nnperformance

net.performFcn = 'crossentropy'; % Cross-Entropy

% Choose Plot Functions

% For a list of all plot functions type: help nnplot

net.plotFcns = {'plotperform','plottrainstate','ploterrhist', ...

'plotconfusion', 'plotroc'};

% Train the Network

[net,tr] = train(net,x,t);

% Test the Network

y = net(x);

e = gsubtract(t,y);

performance = perform(net,t,y)

tind = vec2ind(t);

yind = vec2ind(y);

percentErrors = sum(tind ~= yind)/numel(tind);

% Recalculate Training, Validation and Test Performance

trainTargets = t .* tr.trainMask{1};

valTargets = t .* tr.valMask{1};

testTargets = t .* tr.testMask{1};

trainPerformance = perform(net,trainTargets,y)

valPerformance = perform(net,valTargets,y)

testPerformance = perform(net,testTargets,y)

% View the Network

view(net)

% Plots

% Uncomment these lines to enable various plots.

%figure, plotperform(tr)

%figure, plottrainstate(tr)

%figure, ploterrhist(e)

%figure, plotconfusion(t,y)

%figure, plotroc(t,y)

% Deployment

% Change the (false) values to (true) to enable the following code blocks.

% See the help for each generation function for more information.

if (false)

% Generate MATLAB function for neural network for application

% deployment in MATLAB scripts or with MATLAB Compiler and Builder

% tools, or simply to examine the calculations your trained neural

% network performs.

genFunction(net,'myNeuralNetworkFunction');

y = myNeuralNetworkFunction(x);

end

if (false)

% Generate a matrix-only MATLAB function for neural network code

% generation with MATLAB Coder tools.

genFunction(net,'myNeuralNetworkFunction','MatrixOnly','yes');

y = myNeuralNetworkFunction(x);

end

if (false)

% Generate a Simulink diagram for simulation or deployment with.

% Simulink Coder tools.

gensim(net);

end
